# Composite Detectors Based on Single-Crystalline Films and Single Crystals of Garnet Compounds

**DOI:** 10.3390/ma15031249

**Published:** 2022-02-08

**Authors:** Sandra Witkiewicz-Lukaszek, Vitalii Gorbenko, Tetiana Zorenko, Yurii Syrotych, Jiri A. Mares, Martin Nikl, Oleg Sidletskiy, Pawel Bilski, Akira Yoshikawa, Yuriy Zorenko

**Affiliations:** 1 Institute of Physics, Kazimierz Wielki University in Bydgoszcz, Powstańców Wielkopolskich Stress 2, 85-090 Bydgoszcz, Poland; s-witkiewicz@wp.pl (S.W.-L.); gorbenko@ukw.edu.pl (V.G.); tzorenko@ukw.edu.pl (T.Z.); syr@ukw.edu.pl (Y.S.); sidletskiy@isma.kharkov.ua (O.S.); 2 Institute of Physics, Academy of Sciences of Czech Republic, Cukrovarnicka Stress 10, 16200 Prague, Czech Republic; amares@fzu.pl (J.A.M.); nikl@fzu.pl (M.N.); 3 Institute for Scintillation Materials, National Academy of Sciences of Ukraine, 61072 Kharkiv, Ukraine; 4 Centre of Excellence ENSEMBLE3 Sp. z o.o., ul. Wolczynska 133, 01-919 Warsaw, Poland; 5 Institute of Nuclear Physics, Polish Academy of Sciences, 31-342 Krakow, Poland; pawel.bilski@ifj.edu.pl; 6 Institute for Materials Research, Tohoku University, 2-1-1 Katahira, Aoba-ku, Sendai 980-8577, Japan; yoshikawa@imr.tohoku.ac.jp

**Keywords:** scintillators, thermoluminescence, garnets, crystals, single-crystalline films, substrates, composite detectors, liquid-phase epitaxy

## Abstract

This manuscript summarizes recent results on the development of composite luminescent materials based on the single-crystalline films and single crystals of simple and mixed garnet compounds obtained by the liquid-phase epitaxy growth method. Such composite materials can be applied as scintillating and thermoluminescent (TL) detectors for radiation monitoring of mixed ionization fluxes, as well as scintillation screens in the microimaging techniques. The film and crystal parts of composite detectors were fabricated from efficient scintillation/TL materials based on Ce^3+^-, Pr^3+^-, and Sc^3+^-doped Lu_3_Al_5_O_12_ garnets, as well as Ce^3+^-doped Gd_3__−x_A_x_Al_5__−y_Ga_y_O_12_ mixed garnets, where A = Lu or Tb; x = 0–1; y = 2–3 with significantly different scintillation decay or positions of the main peaks in their TL glow curves. This work also summarizes the results of optical study of films, crystals, and epitaxial structures of these garnet compounds using absorption, cathodoluminescence, and photoluminescence. The scintillation and TL properties of the developed materials under α- and β-particles and γ-quanta excitations were studied as well. The most efficient variants of the composite scintillation and TL detectors for monitoring of composition of mixed beams of ionizing radiation were selected based on the results of this complex study.

## 1. Introduction

The technology of the liquid-phase epitaxy (LPE) developed over the last 30 years offers the possibility of creating luminescent materials on the base of single-crystalline films (SCF) of complex oxides [1,2,3] for application in cathodoluminescent screens [4,5,6], laser media [7,8], scintillators for registration of α- and β-particles, X-rays, or γ-quanta [1,9,10], and scintillating screens for microtomography detectors employing X-ray sources and synchrotron radiation [11,12].

**Figure 1:**
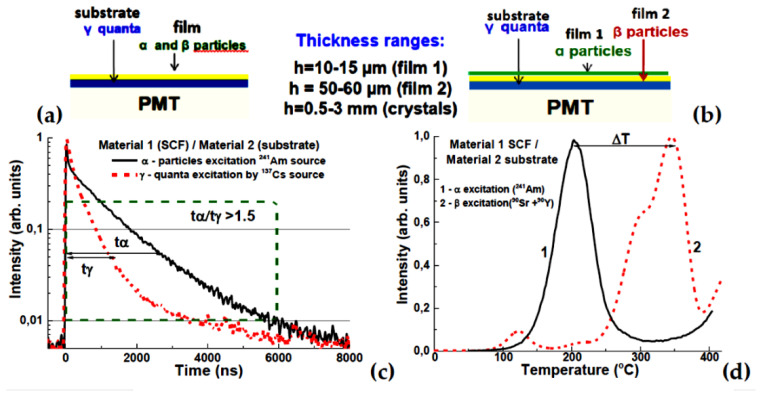
Scheme of a two-layer (**a**) and three-layer (**b**) composite scintillator or TL material, (**c**)—example of the scintillation decay of the film and substrate parts of composite scintillator at registration of α-particles and γ-quanta [14], (**d**)—example of recording ΔT temperature difference between the main peaks in TL glow curves of the film and substrate in a composite TL material [25,26].

Furthermore, advanced composite scintillators [1,9,13,14,15,16,17,18,19,20,21,22] and thermoluminescent (TL) detectors [23,24,25,26] of “phoswich-type” (phosphor sandwich) may be fabricated by the LPE method for simultaneous registration of different components of ionizing radiation, namely, for content analysis of the mixed radiation fluxes involving ionizing particles with various penetration depths. These composite scintillators and detectors comprise epitaxial crystalline structures, including one/two SCFs for registration of low-penetrating α- and β-particles, and bulk single crystal (SC) substrates for registration of the high-penetrating radiation (X- or γ-rays) (Figure 1).

### 1.1. Composite Scintillators and TL Detectors: Fundamentals

The following considerations should be taken into account in development of composite scintillators. The signals from a substrate registering a high-penetrating component of ionizing particles and film registering a low-penetrating component must be distinguished as clearly as possible by luminescence-band spectral composition and/or luminescence decay time (Figure 1a). At the same time, decay times should be fast enough for compatibility with fast operation devices, typically within 1000 ns. Furthermore, light outputs of both film and substrates should be as high as possible to enhance the registration efficiency. A substrate should efficiently absorb, as much as possible, high-penetrating particles, and transmit low-penetrating particles, whereas the opposite is expected from a film. The ideal situation represents a thick-enough substrate comprising a high-density (high-Z_eff_) material, and thin-enough (or low-density/low-Z_eff_) film transmitting a high-penetrating component. The limitations on the materials used in composite scintillators include the misfit between a substrate and film lattice parameters, which should not exceed typically 1–1.3% in the case of garnets [27,28,29], and the component cost, which should be as low as possible by avoiding or minimizing consumption of expensive components such as Lu_2_O_3_ and Ga_2_O_3_. Furthermore, a substrate should be transparent to luminescence of a film if a photodetector is located under the substrate.

Thermoluminescence (TL) is also a well-established method of radiation dosimetry, widely used in radiation protection and medical applications [30,31]. Unlike scintillation, it is a passive integrating technique, in which the measurement of the absorbed dose (‘reading’ of a TL detector) is performed some time (often long) after the actual exposure. The most common TL detectors are those based on lithium fluoride [32,33], but new materials and new applications are still under development [34]. In the past, some types of thin-film TL detectors for measurements of weakly penetrating radiation [35,36,37,38,39] have been developed. Recently, we have also studied the possibility of using LPE-grown SCFs of oxide compounds (perovskites, garnets, orthosilicates) for this purpose [23,24,25,26,39]. Furthermore, the combination of these materials can be considered as well in the frame of development of composite two-layered epitaxial structures capable of registering components in mixed radiation beams (Figure 1b).

Garnets hosts were chosen for development of composites due to their relatively easy growth process in both bulk and film forms. The cubic structure of garnet implies fewer limitations on the film orientation. If considering just “simple” rare-earth garnets, the heaviest Lu_3_Al_5_O_12_ (LuAG) host should be chosen as a substrate [13,14,15,16,17,18,40,41,42,43,44], while the film should comprise lighter Y_3_Al_5_O_12_ (YAG)-based compositions [1,9,13]. The luminescence signals from LuAG and YAG can be distinguished by doping them with different activators having various luminescence lifetimes, such as Pr^3+^ (15–25 ns), Ce^3+^ (40–70 ns), or Sc^3+^ (245–610 ns) in garnet hosts [15,16,40,41,42,43,44], or using the property of faster luminescence decay in films than that in bulk crystals with the same composition due to lower quantity of defects in the former. 

**Figure 2:**
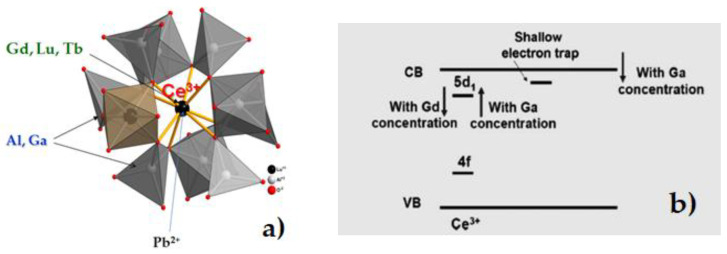
(**a**)—structure of the garnet crystal lattice showing the crystallographic sites occupied by different cations [56]; (**b**)—shifts of 5d_1_ energy level of Ce^3+^ ions in Lu_3−x_Gd_x_Al_5−y_GayO_12_:Ce garnet caused by variations of x and y (CB, VB—conductive and valence bands, respectively) [57,58].

Meanwhile, new prospects in engineering of the garnet composite scintillators have been opened with the development of the bulk crystals of mixed (Lu,Gd,Tb)_3_(Al,Ga)_5_O_12_ garnets possessing much higher light output even over 50,000 photon/MeV [43,44,45]. Nowadays, Ce-doped mixed garnets are among the most extensively developed garnet scintillators [43,44,45,46,47,48,49,50,51,52,53,54,55]. The focus of the recent studies was concentrated on mixed systems with the first (Lu,Gd,Tb) and second (Al,Ga) cation substitutions (Figure 2a). An effect of a nonlinear decrease in the conduction and valence band minima relative to the vacuum referred binding energy with Ga doping in (Lu,Gd,Tb)_3_(Al,Ga)_5_O_12_ garnets was studied in details [43,46]. The change matrix composition also affects the characteristics of Ce^3+^ emission in mixed garnets [43,46,47]. Generally, the substitution of Lu^3+^ cations by the large R = Gd^3+^ or Tb^3+^ ions in the (Lu_x_R_1−x_)_3_Al_5−y_Ga_y_O_12_:Ce lattice results in a red emission shift and in increased splitting of the lowest doublet 5d state of Ce^3+^ with respect to conduction band bottom (Figure 2b), as well as in changing the rates of intra-center and extra-center energy relaxation to Ce^3+^-emitting ions [48,50]. Finally, such double cation substitutions lead to strong increasing LY mixed garnet scintillators with respect to “simple” LuAG:Ce and YAG:Ce garnets [43,50,55].

Furthermore, isomorphic cation substitution in mixed garnets provides also the possibility of precise tuning of lattice parameters of SCF scintillators to reduce a mismatch between films and substrates [59,60,61]. First of all, we considered combinations of the bulk crystals (Gd,Lu)_3_(Al,Ga)_5_O_12_ (GAGG:Ce) scintillators possessing an extremely high light yield and their SCF counterparts [62,63,64,65,66]. Performances of GAGG:Ce substrates and films with varying Al/Ga ratios were explored [62,63,64,65,66]. Finally, experiments on Lu/Tb/Gd and Al/Ga substitutions were conducted to achieve the best optimal and scintillation performance of the composite scintillators [67,68,69].

### 1.2. Composite Scintillators and TL Detectors: History and Perspective

The first composite scintillators, created back in 1990, were based on the LPE-grown Y_3_Al_5_O_12_ (YAG) epitaxial structures [1,9,13,14]. Double-layered YAG:Ce SCF/YAG:Nd SC and YAG:Ce SCF/YAG:Sc SC structures, as well as triply layered composite scintillators of YAG:Ce SCF/YAG:Nd SCF/YAG:Sc SC structure, were LPE-grown and tested under excitations by α- or β-particles and γ-quanta [1,9,13]. The scintillation signals emitted by the SCF and SC parts of composite scintillators were distinguished using the differences in their scintillation decay [1,9,13].

Regarding the radiation monitoring, scintillators with a low density ρ = 4.57 g/cm^3^ and effective atomic number Z_eff_ = 29 based on the YAG SCs were mainly used for low-energy radiation monitoring [1,9,13]. Therefore, garnet composite scintillators with high ρ and Z_eff_ values were demanded for monitoring of mixed fluxes of α- or β-particles and high-energy γ-quanta [14] (Table 1).

Lu_3_Al_5_O_12_ garnet (LuAG) is the most obvious candidate among heavy garnets [11,40,41,42,43,44] with a higher density ρ = 6.73 g/cm^3^ and Z_eff_ = 61 as compared to YAG (Table 1). LuAG:Ce, LuAG:Pr, and LuAG:Sc are well-known scintillators [42,43], while Ce^3+^, Pr^3+^, and Sc^3+^ ions are the most efficient activators in garnet hosts with the different luminescence decay [3,40,41,42,43,44]. It was shown [12,40,41,42,43,44] that the LY of Ce^3+^-, Pr^3+^-, and Sc^3+^-doped LuAG crystals and SCFs exceed those of YAG-based counterparts (Table 1). This became an additional argument for the production of a new generation of high-performance composite scintillators based on LuAG crystals and films [15,16,17,18,19].

Composition engineering of the cation content [42] and bandgap engineering of garnet compounds became a novel approach for development of scintillation materials [57] (Figure 2), opening new avenues for the creation of advanced composite scintillators. Bulk SCs of Gd_3_Al_5−x_Ga_x_O_12_ garnets at x = 2–3 are now on the top list of scintillators with a very high light yield (LY) of up to 50,000 photons/MeV under excitation by γ-quanta of ^137^Cs (662 keV) source [42,43,44,45]. The solid solutions of Lu_3−x_Gd_x_Al_5−y_Ga_y_O_12_ mixed garnets at x = 1–3; y = 2–3 are also very promising materials for creation of the SCF scintillation screens with high absorption ability for X-rays and a very high efficiency of α-particle registration [61,62,63,64,65].

Scintillators in the form of LGAGG:Ce SCFs due to a larger lattice constant can also be crystallized on both YAG and GAGG substrates [61,62,63,64,65]. Furthermore, a successive crystallization of TbAG:Ce SCFs onto undoped GAGG substrates [66,67,68,69], confirmed the possibility of crystallization of good-quality SCF scintillators even in the case of a large (up to 1.3%) misfit between SCF and SC lattices. Following this progress, it seemed reasonable to develop new types of CS for radiation monitoring by combining GAGG:Ce crystals and LGAGG:Ce or TbAG:Ce SCFs into one composite material [20,21,22]. Hence, we developed a novel *approach to the creation of composite scintillators based* on *the combination of different scintillation material hosts doped with the same activator (Ce^3+^).*

Finally, the last part of this review demonstrates the possibility of *developing composite TL detectors based on garnet epitaxial structures.* Among possible compounds, YAG:Ce and LuAG:Ce crystals and SCFs of these garnets, as well as Lu_3−x_Gd_x_Al_5_O_12_:Ce SCFs, were considered [23,24]. The operation principle of such composite TL materials is based on differences in temperatures of main peaks in glow curves corresponding to SCF and SC components under excitation with α- or β-particles (Figure 1b). Therefore, developed garnet epitaxial structures can be considered *as prototypes* of composite TL detectors.

Following the achievements mentioned above, the main goal of our recent activities was crystallization by the LPE method and study of luminescent, scintillation, and TL properties of composite materials based on “film-crystal” epitaxial structures of simple and mixed garnets for monitoring of mixed ionization fluxes in the *active* and *passive* modes based on the scintillation and thermoluminescence phenomena, respectively.

## 2. Materials and Methods

### 2.1. Crystallization of Scintillation Films by LPE Method

**Figure 3:**
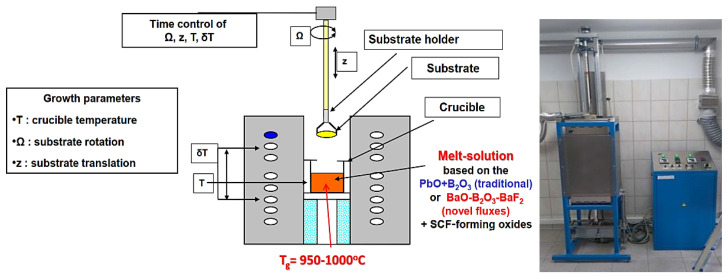
Scheme and photo of the setup for growth of SCF by the LPE method at the Chair of Optoelectronic Materials in the Institute of Physics of University of Kazimierz Wielki in Bydgoszcz.

Composite scintillators based on garnet crystals and films (Figure 3) were grown by the LPE method in the Epitaxy Laboratory of Chair for Optoelectronic Materials in the Institute of Physics of Kazimierz Wielki University (UKW) in Bydgoszcz, Poland. Substrates for film deposition were produced by the Institute for Scintillation Materials (ISMA), Kharkiv, Ukraine (LuAG:Ce, LuAG:Sc, GAGG:Ce), CRYTUR Ltd. Company, Turnov, Czech Republic (LuAG:Pr), and Institute for Materials Research (IMR), Tohoku University, Sendai, Japan (GAGG:Ce).

The LPE method provides crystallization of SCF of different oxide compounds with a very good structure and optical quality with the prescribed thickness. Such a method is based on creation of oversaturation of crystallized substance in solution making it possible to grow films at relatively low temperatures of approximately 1000 °C compared with crystallization conditions at growth of the same materials from the melt at temperatures around 2000 °C.

The charge for the film crystallization is prepared by mixing raw materials containing the film-forming cations in appropriate proportions. In order to calculate the amounts of individual elements needed to obtain the final product, it is necessary to know the molar mass of the raw material, from which the element can be selected in the respective proportion.

A standard purity of raw material components was not less than 99.99%. During the preparation of the charge for the production of thin SCFs using the LPE method, the so-called Blank–Nielsen coefficients $R_{1}$, $R_{2}$, $R_{3}$ i $R_{4}$ should be maintained corresponding to the ratios:

$$R_{1}=\frac{P_{flux_{PbO}}}{P_{flux_{B2O3}}};\;R_{2}=\frac{\sum P_{garnet\left(dod\right)}}{\sum P_{garnet\left(oct+tet\right)}};\;R_{3}=\frac{\sum P_{garnet}}{\sum P_{garnet}+\sum P_{flux}};\;R_{4}=\frac{\sum P_{dopant}}{\sum P_{garnet}}\;,$$

where *P* are mole weights of the PbO and B_2_O_3_ flux components as well as garnet and activator host components occupying dodecahedral (dod), octahedral (oct), and tetrahedral (tet) positions in the garnet lattice, respectively.

The ratio *R*_1_ = 11–12 determines kinetic characteristics of the solution and the solubility of the oxides constituting the film. *R*_2_ = 0.02–0.035 corresponds to the garnet phase as the main phase at the film crystallization. Meanwhile, the choice of *R*_3_ and *R*_4_ in the ranges of 0.02–0.035 and 0.01–0.15, respectively, relates to the optimization of a film scintillation efficiency.

The charge was dissolved in a mix of lead oxide PbO and boron oxide B_2_O_3_ in the ratio of 88–90%: 10–12%, which was typically used as a flux. The molar concentration of the flux in the solution expressed by the *R*_3_ coefficient was 95–97%. Flux components, in particular, divalent lead ions Pb^2+^ and tetravalent Pb^4+^ ions, may also incorporate the films and give rise to undesirable effects. Lead admixture strongly reduces the luminescence efficiency of activators such as Ce^3+^, Pr^3+^, and Sc^3+^. On the other hand, solutions of PbO and B_2_O_3_ are characterized by a very good solubility of film-forming materials, relatively low viscosity, and high fluidity that is highly important during crystallization of films with a high structural and optical quality.

The prepared raw materials with the composition meeting the specified *R*_1_–*R*_4_ values were placed in Pt crucibles of 30–40 mm diameter. The crucible material was chosen accounting for a high melting temperature of 1768 °C and weak impact of Pt admixture on optical quality of films.

The basic mechanism determining the formation of a single-crystalline film onto the substrate is the process of *supercooling the solution*. For this purpose, a crucible is placed in a furnace (Figure 3) and heated to a temperature of 1050–1100 °C. At this temperature, the melted materials form an unsaturated solution characterized by a certain saturation temperature *T_S_* (*solidus temperature*), which is a function of the *R*_3_ coefficient. As solution temperature is lowered below *T_S_* to growth temperature *T_g_* in the 950–1050 °C range, the solution undercools, and the excess solute deposits on the rotating substrate introduced into the solution.

The substrate dimensions should not exceed half of the crucible diameter, i.e., 15–20 mm. The SCF growth rate of depends on several factors, mainly, the difference Δ*T* between growth temperature *T_g_* and melt saturation temperature *T_S_*. In general, the film thickness is proportional to the degree of supercooling Δ*T = T_g_ − T_S_*, and the square root of the substrate rotation rate ω. The obtained SCF scintillators typically had thickness in the 15–50 µm range. The LPE growth of SCFs of multi-component mixed garnets, in general, is complicated due to chemical complexity and specific segregation phenomena for different types of cations [59,60,61,62,63,64,65]. For this reason, typically the actual SCF composition differs with respect to the nominal melt-solution composition.

### 2.2. Characterization of Composite Scintillators and TL Detectors

The actual compositions of single crystals and films was determined using a JEOL JSM-820 (JEOL Ltd., Akishima, Japan) electronic microscope equipped with an IXRF 500 i LN2 Eumex EDX detector (IXRF, Inc., Austin, TX, USA). Structural quality of SCFs with different content, as well as the SCF/substrate misfit **m** = [(**a_f_** − **a_sub_**)/**a_sub_**] × 100% between the lattice constant of SCF **a_f_** and substrate **a_sub_**, were determined from XRD patterns (diffractometer DRON 4-07 with Cu_Kα_ X-ray source, Boureviestnik, S.-Petersburg, USSA-Russia).

Absorption spectra, cathodoluminescence (CL) spectra, light yield (LY), and scintillation decay kinetics were measured under excitation by α-particles from ^239^Pu (5.15 MeV) and ^241^Am (5.5 MeV) sources as well as ^137^Cs (662 keV) source, respectively. Gd_3_Al_2.5_Ga_2.5_O_12_:Ce (GAGG2.5:Ce) and Gd_3_Al_2_ Ga_3_O_12_:Ce (GAGG3:Ce) crystals with the size of 5 × 5 × 0.9 mm produced in ISMA, Ukraine and IMR Tohoku University, Japan, as well as YAG:Ce SCF, were used as reference samples at composite scintillators characterization. The absorption spectra were registered using a Jasco 760 UV-Vis spectrometer (Jasco, Easton, USA) in the 200–1100 nm range. The CL spectra were registered at room temperature (RT) using an SEM JEOL JSM-820 electron microscope (JEOL Ltd., Akishima, Japan) equipped with a Stellar Net spectrometer and TE-cooled CCD detector working in the 200–925 nm range. The scintillation LY determined from pulse height spectra (PHS) recorded with a shaping time of 12 μs was registered using a setup based on a Hamamatsu H6521 photomultiplier (Hamamatsu Photonics K.K., Japan) (PMT), multi-channel analyzer, and a Tektronix TDS3052 digital oscilloscope (Tektronox, INC., Beaverton, Oregon, USA) under excitation by α-particles of ^239^Pu (5.15 MeV) source. The spectra were calibrated with a standard YAG:Ce SCF sample with a photoelectron yield of 360 phels/MeV and LY of 2650 photons/MeV.

Scintillation response of composite scintillators was determined using the setup consisting of a hybrid PMT (HPMT DEP PP0475B), controlled by PC. PHS were registered under excitation with α-particles with the energy of 5.4857 MeV of ^241^Am radioisotope and with γ-rays of ^137^Cs (energy 661.66 keV) radioisotope. Herein, α-particles of ^239^Pu and ^241^Am sources excite only epitaxial layers of SCF samples (not their substrates), because α-particle penetration depth in the studied materials is approximately 12–15 μm.

TL glow curves were measured under excitation by α- and β-particles from ^241^Am and ^90^Sr + ^90^Y sources using a Risø TL/OSL-DA-20 reader (Risø DTU, Roskilde, Denmark). A “green” Schott BG 39 filter was used for the registration of TL signal. The filter transmittance range of 350 to 700 nm matched well with the Ce^3+^ emission range.

## 3. Results

### 3.1. Composite Scintillators Based on LuAG Substrates Doped with Pr, Sc, and Ce Ions

#### 3.1.1. Substrates Based on LuAG Crystals

**Figure 4:**
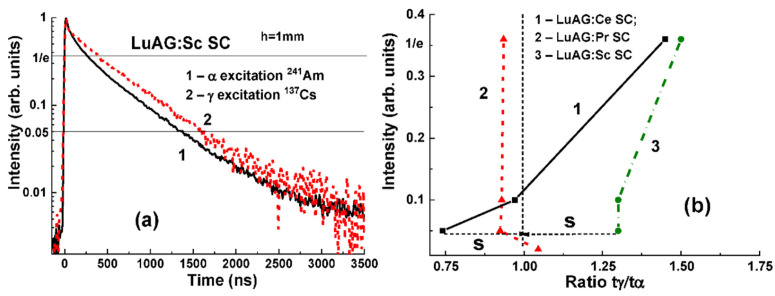
(**a**)—scintillation decay kinetics of LuAG:Sc substrate under with α-particles (1) and γ-quanta excitations (2); (**b**) t_γ_/t_α_ ratio at different luminescence intensities for LuAG:Ce (1), LuAG:Pr (2), and LuAG:Sc (3) substrates [27].

In the first phase of composite scintillator development, it is very important to analyze the scintillation decay curves of Ce^3+^-, Pr^3+^-, and Sc^3+^-doped LuAG substrates under excitation with α-particles and γ-quanta. This analysis was performed on 1 mm-thick substrates (Figure 4).

The various scintillation decays for the mentioned substrates are illustrated by Figure 4a. Slightly larger difference is observed for the LuAG:Ce and LuAG:Sc SC substrates, which can be quantified by the t_γ_/t_α_ ratio reaching 1.45 and 1.5, respectively, on the 1/e level.

#### 3.1.2. Composite Scintillators Based on LuAG:Ce Substrates

**Figure 5:**
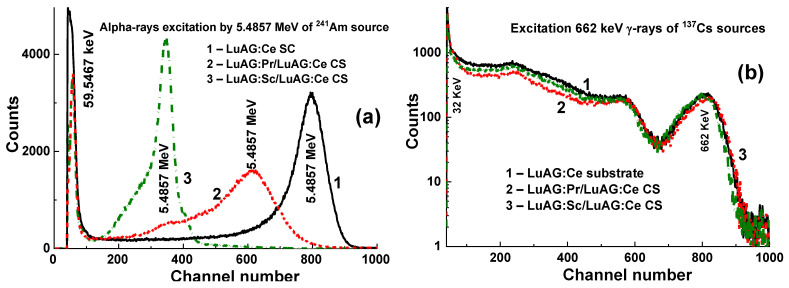
PHS of LuAG:Ce SC substrate (1), LuAG:Pr SCF/LuAG:Ce SC (2), LuAG:Sc SCF/LuAG:Ce SC, (3) composite scintillators under α-particles (**a**) and γ-quanta (**b**) excitations [15,16].

LuAG:Pr SCF/LuAG:Ce and LuAG:Sc SCF/LuAG:Ce composite scintillators were developed in [15,16]. Features of α-particle and γ-quantum interaction with the mentioned materials cause a big difference in scintillation efficiency at their registration, expressed by the ratio LY_α_/LY_γ_. Figure 5 shows PHS of these composite scintillators under excitation by α-particles and γ-quanta. The main peak in Figure 5a corresponds to the full absorption of α-particles with a 5.5 MeV energy, while the left peak is associated with the absorption of γ-quanta of a ^137^Cs source with an energy of 59.65 keV. It is important that the positions of the peaks corresponding to α-particle excitation of LuAG:Pr and LuAG:Sc SCFs are different from those for the LuAG:Ce SC substrate (Figure 5a). This means that α-particles are registered only by the SCF and do not excite the substrate.

At excitation of LuAG:Pr SCF/LuAG:Ce SC and LuAG:Sc SCF/LuAG:Ce composite scintillators by γ-quanta from ^137^Cs source, the additional and main peaks in the amplitude spectra correspond to the total absorption of γ-rays with the 32 keV and 662 keV energies (Figure 5b). It is important that the main photopeaks of LuAG:Ce SC and composite scintillators in Figure 5b have a similar position certifying full absorption of γ-quanta in the substrate parts of such composites.

**Figure 6:**
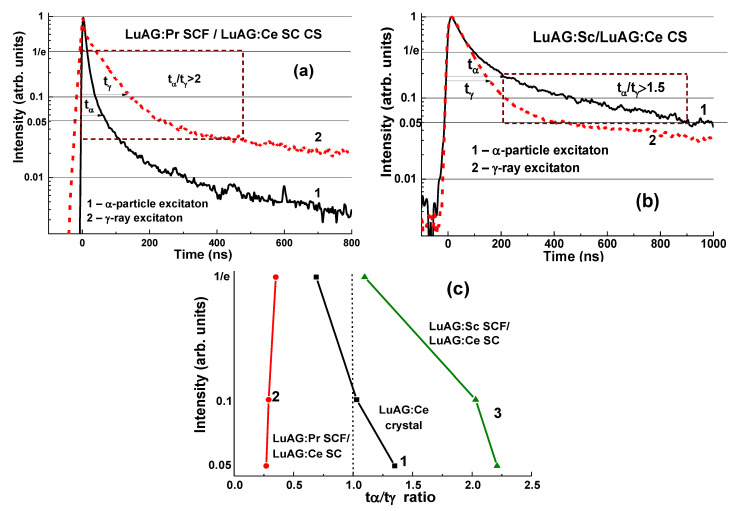
Scintillation decay kinetics of LuAG:Pr SCF/LuAG:Ce SC (**a**) and LuAG:Sc SCF/LuAG:Ce SC (**b**) composite scintillators under excitation by α-particles (1) and γ-quanta (2). (**c**)—t_γ_/t_α_ ratio for different levels of scintillation decay for these CS (1, 2) compared to the LuAG: Ce substrate (3) [15,16].

The remarkable differences in the scintillation decay of LuAG:Pr SCF/LuAG:Ce SC and LuAG:Sc SCF/LuAG:Ce SC composite scintillators are observed under γ-rays and α-particles with a t_α_/t_γ_ value ranging within 0.27–0.35 and 1.1–2.2, respectively, at luminescence-intensity decay from 1/e down to 0.05 (Figure 6c) [15,16].

A LuAG:Pr SCF/LuAG:Ce SC composite scintillator has a certain advantage over LuAG:Sc SCF/LuAG:Ce SC composition due to a larger t_α/γ_ ratio in the entire 0–700 ns time range (Figure 6a). However, the LuAG:Sc SCF/LuAG:Ce SC composite scintillator separates the scintillation signals from the SCF and the substrate even with a better t_α_/t_γ_ ratio, but in the narrow time interval of 200–900 ns and in a smaller intensity range between 0.2 and 0.05 (Figure 6c). Meanwhile, these results prove that both types of composite scintillator are capable of distinguishing α-particles and γ-quanta in mixed fluxes.

#### 3.1.3. Composite Scintillator Based on LuAG:Pr Substrates

This subsection is focused on the development of composite scintillators based on the LuAG:Pr SC and SCF of Lu_2−x_GdTb_x_Al_5_O_12_:Ce and Lu_3−x_Tb_x_AG:Ce mixed garnets with an *x* range of 0.15–2.285. Adjusting the cation ratio in the garnet compositions provides favorable changes in scintillation properties of composite scintillators [17].

**Figure 7:**
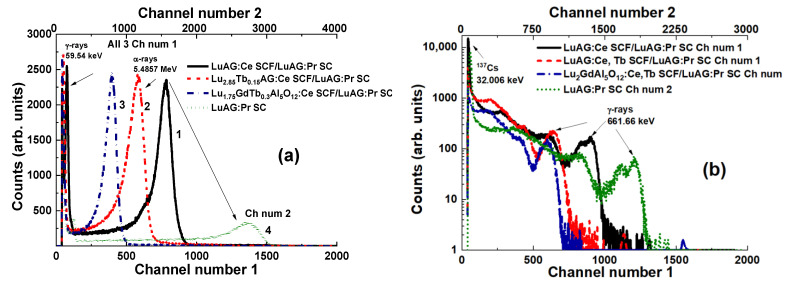
PHS of LuAG:Ce SCF/LuAG:Pr SC (1), Lu_2.85_Tb_0.15_AG:Ce SCF/LuAG:Pr SC (2), Lu_1.7_GdTb_0.3_AG:Ce SCF/LuAG:Pr SC (3) composite scintillators and LuAG:Pr SC (4) under excitation with α-particles (**a**) and γ-quanta (**b**) [17].

Amplitude spectra of LuAG:Ce SCF/LuAG:Pr SC, Lu_2.85_Tb_0.15_AG:Ce SCF/LuAG:Pr SC, and Lu_1.7_GdTb_0.3_AG:Ce SCF/LuAG:Pr SC composite scintillators for the registration of α-particles and γ-quanta are presented in Figure 7. Different locations of the SCFs’ main peaks in the mentioned composite scintillators compared to the LuAG:Pr substrate indicate that α-particles excite only the SCF part of the composite scintillator. The same situation is observed under excitation of the mentioned composites with γ-quanta (Figure 7b). The SCF main peak location depends on the type, thickness, and LY of composite scintillators (Figure 7a) and evidences the significant impact of LuAG-based SCF to the γ-ray absorption.

The best separation of the decay curves from the LuAG:Pr substrate and Lu_3−x_Tb_x_AG:Ce SCF at x = 0.15–0.3 was achieved at high concentration of Tb^3+^ cations (x = 1.65–2.285). For comparison, the scintillating decay times of Lu_3−x_ Tb_x_AG:Ce (x = 0.64; 1.05 and 2.15) SCFs scintillators grown onto LuAG:Pr substrates were compared under α-particle and γ- ray excitations. Crystallization of the latter, however, was associated with a high mismatch of crystal lattices above 1%.

**Figure 8:**
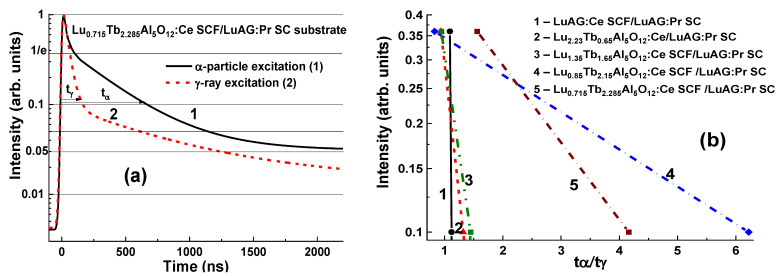
(**a**)—scintillation decay of Lu_0.715_Tb_2.285_Al_5_O_12_:Ce SCF/LuAG:Pr SC composite scintillator (**b**) under excitation by α-particles (1) and γ-quanta (2). (**b**)—t_γ_/t_α_ ratios for the different levels of luminescence-intensity decrease for Lu_3−x_Tb_x_Al_5_O_12_:Ce SCF/LuAG:Pr SC composite scintillators with the Tb content x = 0 (1), 0.65 (2), 1.65 (3), 2.15 (4), and 2.285 (5) [17].

Following the successful crystallization of Lu_3−x_ Tb_x_AG:Ce SCF with Tb x = 0.65–2.285 onto LuAG:Pr substrates, remarkably, better t_γ_/t_α_ values were obtained in the composite scintillators with high Tb concentrations (Figure 8b). An example of a good separation of the decay curves at excitation of the Lu_0.715_Tb_2.285_AG:Ce SCF/LuAG:Pr SC composite scintillator with α-particles and γ-quanta presented in Figure 8a demonstrates the t_α_/t_γ_ ratios in the range of 1.56–4.16 as the luminescence decays from 1/e to 0.1 (Figure 8b).

#### 3.1.4. Composite Scintillators Based on LuAG:Sc Substrate

**Figure 9:**
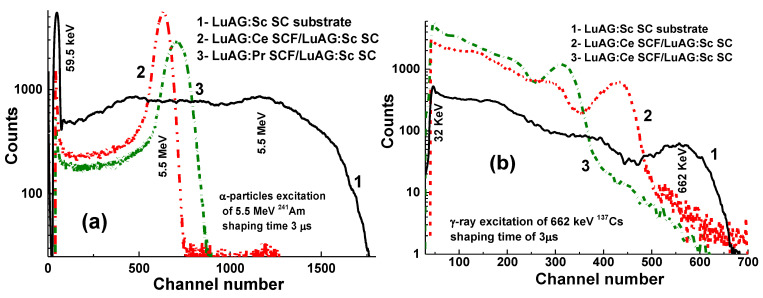
Pulse height spectra of LuAG:Ce SCF/LuAG:Sc SC (1) and LuAG:Pr SCF/LuAG:Sc SC (2) composite scintillators, and LuAG:Sc substrate (3) measured in a time range of 3 µs at α-particle (**a**) and γ-quanta (**b**) excitation [27].

The last set of composite scintillators based on LuAG epitaxial structures are LuAG:Ce and LuAG:Pr SCFs with 12–30 µm thickness based on LuAG:Sc substrates [18,19]. PHS of these composite scintillators under excitation by α-particles and γ-quanta are presented in Figure 9. It is noteworthy that, at registration of α-particles, the main peaks are shifted relative to each other and to the substrate (Figure 9a), because α-particles excite only the film part of composite scintillators. At registration of γ-quanta, the main peaks are also shifted (Figure 9b), indicating the excitation of both the substrate and SCF. Therefore, the total absorption of 662 keV γ-quanta also depends on the type, thickness, and LY of the SCF affecting scintillation properties of the entire composite scintillators.

**Figure 10:**
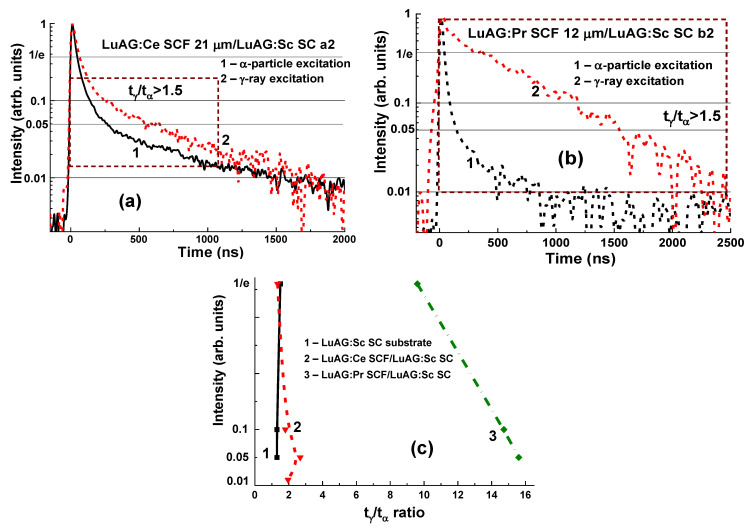
Scintillation decay of LuAG:Ce SCF/LuAG:Sc SC (**a**) and LuAG:Pr SCF/LuAG:Sc SC (**b**) composite scintillators under excitation with α-particle (1) and γ-quanta; (**c**)—t_γ_/t_α_ ratio for different levels of scintillation decay of LuAG:Ce SCF/LuAG:Sc SC (2) and LuAG:Pr SCF/LuAG:Sc SC (3) composite scintillator compared to that in LuAG:Sc substrate (1) [18].

For LuAG:Ce SCF/LuAG:Sc SC and LuAG:Pr SCF/LuAG:Sc SC composite scintillators, the differences in decay times at different levels of the luminescence decay (1/e, 0.1, 0.05, and 0.01) under excitation by α-particles and γ-quanta are shown in Figure 10. The selection of samples with different SCF thicknesses in the 12–30 µm range also enables analyzing of the effect of this factor on the separation of decay curves at different types of excitation. The best signal separation from the SCF and SC over the entire time range was achieved in LuAG:Pr SCF/LuAG:Sc SC structure with an SCF thickness of 12 µm and a 1 mm-thick substrate (Figure 10b). The t_γ_/t_α_ ratios range from 9.6 to 15.6 at luminescence-intensity decrease from 1/e to 0.05, which is the best result among all the developed types of composite scintillators based on LuAG epitaxial structures doped with Ce^3+^, Pr^3+^, and Sc^3+^ ions.

The LuAG:Ce SCF/LuAG:Sc SC composite scintillator also separates α-particles and γ-quanta (Figure 10c) with t_γ_/t_α_ in the range of 1.34–1.96 at the scintillation decay from 1/e to 0.01, which is low compared to that in bare LuAG:Sc substrate (Figure 4a and Figure 10c).

### 3.2. Composite Scintillators Based on Gd_3_Al_2.5_Ga_2.5_O_12_:Ce Substrates

#### 3.2.1. Characterization of GAGG:Ce Substrates

Successful crystallization of SCFs and bulk ingots of mixed garnets (Lu,Gd,Tb)_3_(Al,Ga)_5_O_12_:Ce opens new avenues in the engineering of new types of film-substrate composite scintillators. Firstly, lower density and Z_eff_ of mixed garnet SCF yields a lower ability to absorb γ-quanta compared to LuAG that should provide better signal separation under mixed radiation fluxes. Secondly, Gd_3_Al_5−x_Ga_x_O_12_ (x = 2–3) garnets are characterized by a very high LY under γ-quanta (^137^Cs, 662 keV) up to 50,000 photon/MeV [42] compared to LuAG:Ce, LuAG:Pr, and LuAG:Sc substrates. For these reasons, SCFs and SC of mixed garnets are a good choice for enhanced composite scintillators.

**Figure 11:**
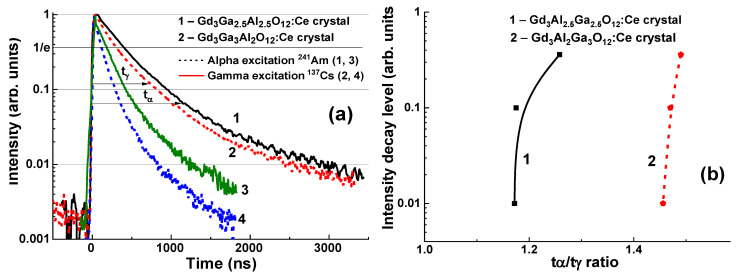
Scintillation decay curves of GAGG2.5:Ce and GAGG3:Ce crystals under α-particle excitation (curve 1) and γ-ray excitation (curve 2) (**a**). (**b**) t_γ_/t_α_ ratio of scintillation decay to 1/e, 0.1, 0.05, 0.01 levels for GAGG2.5:Ce (1) and GAGG3:Ce 3.0 (2) crystals [20,22].

There were two types of the available Gd_3_Al_5−x_Ga_x_O_12_:Ce substrates with x = 2.5 and 3 and a thickness of 1 mm. The scintillation decay curves of these substrates under excitation by α-particles and γ-quanta are presented in Figure 11. It was observed that the scintillation decay of substrates under γ-quanta excitation is systematically faster than that under α-particles, which is caused by a specific interaction of these radiations with the scintillator material. The difference in light yield of the GAGG:Ce substrates at these excitation types expressed by the LY_α_/LY_γ_ ratio is in the range of 0.195–0.2 [20,22].

As one may see in Figure 11a, an increase in the Ga concentration from x = 2.5 to 3 leads to a significant acceleration of scintillation decay for both excitation types, as well as better separation of the scintillation decay under α-particles and γ-quanta, with the t_γ_/t_α_ ratio of 1.46–1.49 at scintillation decay from 1/e to 0.01 as compared to 1.17–1.26 for GAGG:Ce with x = 2.5 (Figure 11b).

#### 3.2.2. Composite Scintillators Based on the GAGG:Ce Crystals and SCF

The results of previous research of the scintillation properties of Gd_3_Al_5−x_Ga_x_O_12_:Ce (GAGGx:Ce) SCs and SCFs [64,65] open wide possibilities for development of *new types of composite scintillators based on the mixed garnets with different Ga^3+^ concentration*. Scintillation decay kinetics of SCF and single crystals parts of composite scintillators based on the mentioned mixed garnets can be specially optimized and fitted due to different Ga content [16,20,22]. This will lead to better separation of scintillation signals for the detection of different types of ionizing radiation.

Strong modification of the scintillation decay kinetics of SCF doped with Ce^3+^ LuAG and mixed grenades is observed due to co-doping with M^2+^ (M = Mg^2+^, Ca^2+^) and formation of partial cerium ions in the Ce^4+^ charge state [70,71,72,73,74]. In this way, the scintillation decay kinetics of the doubly doped garnet compounds of Ce^4+^-M^2+^ garnet are strongly accelerated. However, the LY of these SCF scintillators is significantly reduced due to the lower scintillation efficiency of Ce^4+^-M^2+^ centers compared to the “conventional” Ce^3+^ center. In our opinion, however, such an approach may also be interesting in the case of creating composite scintillators based on simple or mixed compounds of garnets with a relatively similar cation content.

In this part of the report, we present the results of the development of new types of composite scintillators based on SC and SCF of Ce^3+^-doped GAGGx:Ce garnet with different Ga x concentration by LPE growth method [22].

**Figure 12:**
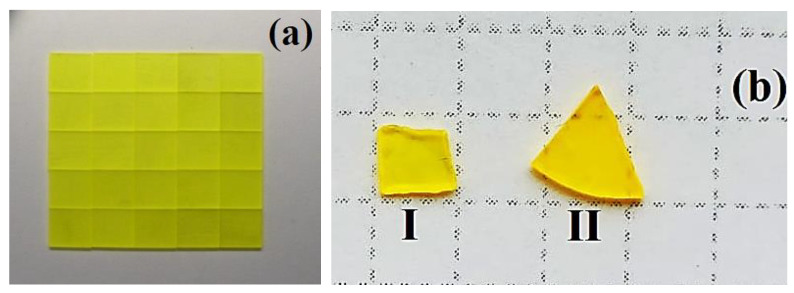
Substrates prepared from GAGG2.5:Ce crystal (**a**) and two composite scintillators based on Gd_3_Al_3_Ga_2_O_12_:Ce SCF (GAGG2:Ce) (**b**I) and Gd_3_Al_2_Ga_3_O_12_:Ce (GAGG3:Ce) (**b**II) SCF grown onto GAGG2.5:Ce substrates [22].

The real compositions of GAGG:Ce SCs and SCFs (Figure 12) are presented in Table 2. According to these results, the Ga ion segregation coefficient in Gd_3_Al_5−x_Ga_x_O_12_:Ce SCFs grown on GAGG2.5:Ce SC melt—solution at x = 2–4 *is equal to 0.58–0.65*. For this reason, the real x concentration of Ga in the SCF samples GAGG2:Ce, GAGG3:Ce, GAGG3.5:Ce, and GAGG4:Ce SCF is 1.16, 1.67, 2.17, and 2.615, respectively.

Overall, the LY in Gd_3_Al_5−x_Ga_x_O_12_:Ce (x = 2–4) SCFs decreases with Ga content (Table 2). Namely, with excitation of the α-particles by the ^239^Pu source (5.15 MeV), it is 2–3 times lower than in the reference YAG:Ce SCF sample and more than 5–10 times lower compared to the GAGG:Ce substrate. This phenomenon is caused by a higher concentration of Pb^2+^ flux and lower Ce^3+^/Pb^2+^ ratios in Gd-rich SCF samples compared to YAG:Ce SCF due to the increase in the lattice constant, namely, the volume of dodecahedron sites for localization of relatively large Pb^2+^ ions [22,23].

**Figure 13:**
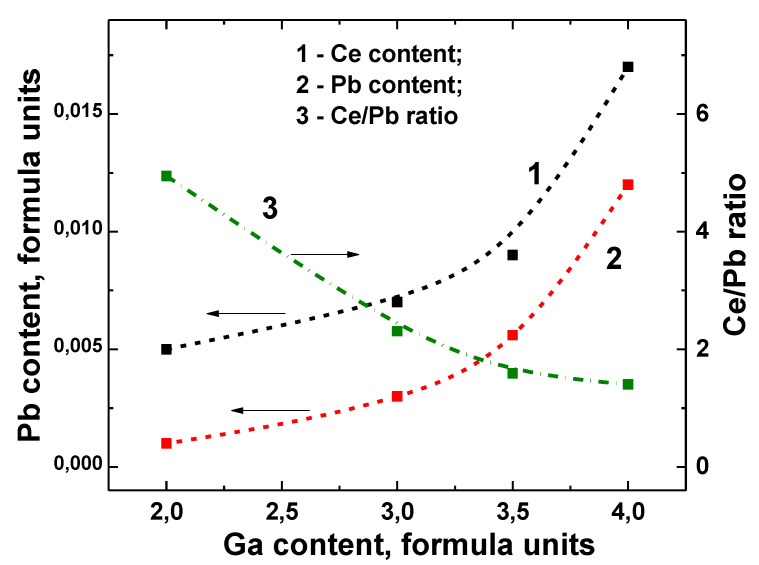
Dependence of Ce (1) and Pb (2) concentration, as well as Ce/Pb ratio (3) in Gd_3_Al_5−x_Ga_x_O_12_:Ce SCFs, on nominal Ga content x in melt-solution [22].

It is well known that the Pb^2+^ ion is a very effective quencher of Ce^3+^ luminescence in garnets and other oxide compounds, negatively affecting their scintillation properties [3,22,27,28,29,64]. The increase in Pb^2+^ contamination and Ce^3+^ concentrations in GAGG:Ce SCFs is stimulated by an increase in the dodecahedral site volume for localization of the relatively large Pb^2+^ ions [22,59,60,62,63,64]. Indeed, as can be seen in Table 2 and Figure 13, the lead content of Gd_3_Al_5−x_Ga_x_O_12_:Ce SCFs is relatively high and increases steadily with Ga content. For this reason, the Ce^3+^/Pb^2+^ ratio in Gd_3_Al_5−x_Ga_x_O_12_:Ce SCFs is 1.4–5, while typical values are 15–17 (Figure 13) [22,62,63,64].

**Figure 14:**
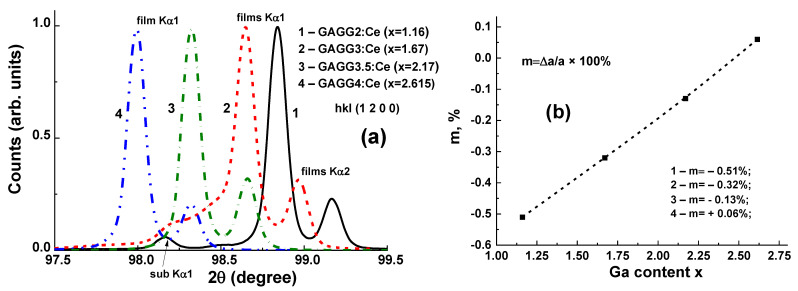
(**a**)—XRD of Gd_3_Al_5−x_Ga_x_O_12_:Ce SCFs grown from melt solution with the nominal Ga content x in the 2–4 range and real Ga content in the x = 1.16–2.615 range; (**b**)—dependence of misfit *m* on Ga content x in SCF samples [22].

The structural quality of Gd_3_Al_5−x_Ga_x_O_12_:Ce SCFs with different Ga concentrations was studied, as well as the lattice constant of SCF samples and the misfit between the lattice constants of SCFs and GAGG:Ce substrate Δa = (a_SCF_–a_sub_)/a_sub_*100% (Figure 14). The lattice constant of Gd_3_Al_5−x_ Ga_x_O_12_:Ce SCFs in the 12.168–12.235 Å range and *m* value in the −0.51% < *m* < +0.06% range depend linearly on the Ga x content in these samples. It should be noted that such small mismatch values are very suitable for the deposition of SCF scintillators with high structural and optical quality.

**Figure 15:**
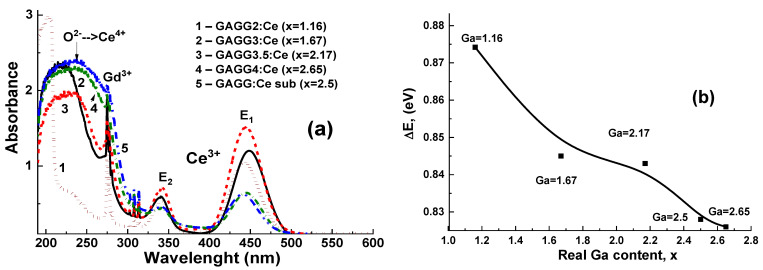
Absorption spectra (**a**) of epitaxial structures, containing Gd_3_Al_5−x_Ga_x_O_12_:Ce (x = 2–4) SCFs, grown onto GAGG2.5:Ce substrates (2–5) in comparison with absorption spectra of GAGG:Ce 2.5 substrate (curve 1). Shift of the absorption bands E1 and E2 in Ce^3+^ related to Ga content (**b**) [22].

The absorption spectra of Gd_3_Al_5−x_Ga_x_O_12_:Ce/GAGG2.5:Ce (x = 1.16–2.615) composite scintillators compared to the spectra of the GAGG2.5:Ce substrate are shown in Figure 15. It is worth noting that the absorption spectra of composite structures are a superposition of the spectra of the GAGG:Ce substrate and two SCF samples on either side of these substrates. The sharp bands peaked at 275 and 313 nm in the substrate spectrum and all SCFs attributed to the absorption band of Gd^3+^ ions strongly overlap with the absorption bands peaking in the 260–265 nm range, caused by the intrinsic ^1^S_0_→^3^P_1_ transitions in Pb^2+^ [22,62,63].

The wide absorption bands of Gd_3_Al_5−x_Ga_x_O_12_:Ce/GAGG2.5:Ce composite scintillators with different Ga concentration in SCF samples and GAGG2.5:Ce substrate in the ranges of 339–341 nm and 440–450 nm (marked as E2 and E1 bands, respectively) are associated with the 4f–5d (^2^E) transitions of Ce^3+^ ions. The remaining Ce^3+^ absorption bands in these scintillators are below 230 nm and are related to the 4f–5d (T_2g_) transitions [62,63]. As the Ga content increased in the range of 1.16–2.65 in Gd_3_Al_5−x_Ga_x_O_12_:Ce SCF, we observed a shift of the absorption bands E_1_ and E_2_ in Ce^3+^ (Figure 15a), and a change in the corresponding values of ΔE = E_2_ − E_1_ (Figure 15b). The value of ΔE is the largest in the composite scintillator sample with the smallest Ga content in SCF (x = 1.16), and it systematically decreases with increasing Ga concentration in SCF, because Ga concentration approaches the gallium concentration x = 2.5 in GAGG substrate: Ce (Table 2).

The absorption spectra of Gd_3_Al_5−x_Ga_x_O_12_:Ce/GAGG2.5:Ce (x = 1.16–2.615) composite scintillators show the presence of an additional broadband peak at about 255 nm (Figure 15). This band is related to the O^2+^→Ce^4+^ charge transfer transitions (CTT) (see [70,71,72,73,74] for details). The formation of Ce^4+^ states in these SCFs, especially in the samples with the highest Ga content, is due to the inclusion of the impurity associated with the Pb^2+^ flux (Table 2). Indeed, the intensity of O^2+^→Ce^4+^ CTT band peaked at 255 nm systematically increases in the absorption spectra of Gd_3_Al_5−x_ Ga_x_O_12_:Ce SCF/GAGG2.5:Ce composite scintillators as Ga content x rises from 1.16 to 2.615 in SCF (Figure 15). Such a phenomenon correlates well with a remarkable increase in Pb^2+^ concentration in Ga-rich SCF samples (see Table 2).

The content of large Pb^2+^ ions in SCF samples with the largest Ga concentration may be contributed to the decrease in their LY (see Table 2). A negative influence of Pb^2+^ on the LY of epitaxially grown films was observed in many scintillators [3,22,27,28,29,62,63,64]. Such influence can be attributed to the creation of Pb^2+^-Ce^4+^ pairs due to charge and volume compensation at relatively large concentration of lead ions in SCF samples and subsequent reduction in Ce^3+^ concertation ions in garnet hosts (see [70,71,72,73] for details).

**Figure 16:**
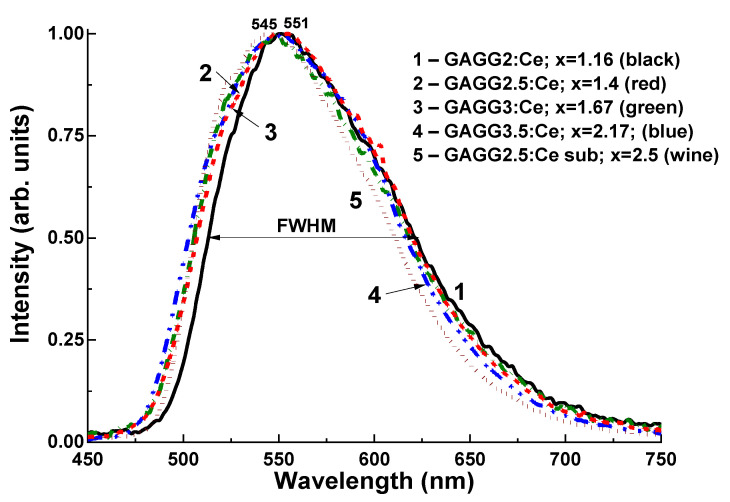
Normalized CL spectra of Gd_3_Al_5−x_Ga_x_O_12_:Ce (x = 1.16–2.17) SCFs in comparison GAGG2.5:Ce substrate (5) [29].

Normalized CL spectra of the Gd_3_Al_5−x_Ga_x_O_12_:Ce SCF part of composite scintillators with the range x = 1.16–2.16 and the substrate GAGG2.5:Ce are shown in Figure 16. The CL spectra of all SCFs and the substrate show only the wide luminescence of the Ce^3+^ ion band in the visible range. They peaked at 545–551 nm assigned to the 5d^1^→4f (^2^F_5/2;7/2_) transitions of the Ce^3+^ ion in the mentioned garnet hosts. Ce^3+^ luminescence peaks in Gd_3_Al_5−x_Ga_x_O_12_:Ce SCFs are clearly shifted in the blue direction, and the FWHM of these bands increases with increasing Ga content in SCF in the range x = 1.16–2.16. These trends are well consistent with the results of similar studies of the CL Gd_3_Al_5−x_Ga_x_O_12_:Ce SCFs spectra disclosed in [62,63,64] and can be explained by the decreasing crystal field strength at the dodecahedral position of the respective garnet hosts. An exception is the CL spectrum of the GAGG2.5:Ce substrate, which shows a lower FWHM than the corresponding SCF (Figure 16, curve 5). However, this effect is due to the strong reabsorption of the high-energy wing of the emission band with the corresponding absorption band 4f–5d of Ce^3+^ ions with a peak of 442 nm in a thick crystalline substrate compared to that in thin SCF with similar compositions.

**Figure 17:**
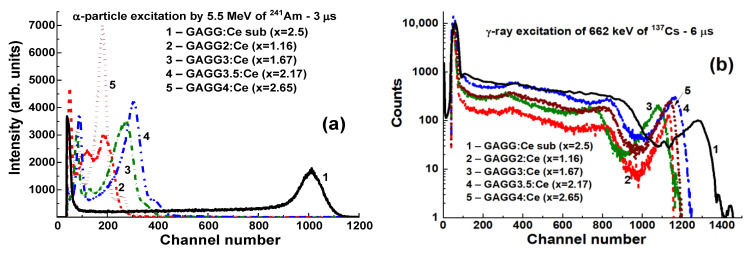
PHS of Gd_3_Al_5−x_Ga_x_O_12_:Ce SCF/GAGG:Ce SC composite scintillators (2–5) with different Ga content x = 1.6–2.67 in SCFs (2–5) and GAGG:Ce substrate (1) measured under α-particle excitation with energies of 59.6 keV and 5.5 MeV of ^241^Am source (**a**) and γ-excitation with an energy of 662 keV of ^137^Cs source (**b**) [22].

The PHS registered with Gd_3_Al_5−x_Ga_x_O_12_:Ce SCF/GAGG2.5:Ce SC composite scintillators with Ga concertation in the SCFs in the x = 1.16–2.65 range under excitation by α-particles and γ-rays of ^137^Cs source are presented in Figure 17a,b, respectively. The main peaks in Figure 17a correspond to the total absorption of α-particles, while the peaks in the left part of the spectra are related to the absorption of the low-energy emission from ^241^Am source.

**Figure 18:**
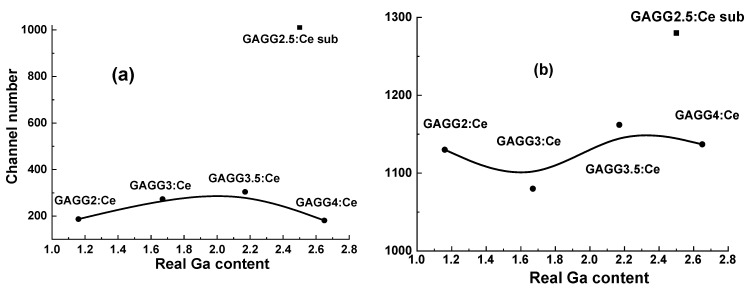
Location of the main photopeak of Gd_3_Al_5−x_Ga_x_O_12_:Ce SCF/GAGG2.5:Ce SC composites and GAGG2.5:Ce SC substrate under excitation by α-particles (**a**) and γ-quanta (**b**) [22].

It should be noted that the positions of the main photopeaks of the source of the ^241^Am α-particles observed in Figure 17a and Figure 18a are significantly different for all Gd_3_Al_5−x_Ga_x_O_12_:Ce SCF/GAGG:Ce SC composites and the GAGG2.5:Ce substrate. This means that *the α-particles excite only parts of the SCF of the composite scintillators*. The largest scintillation efficiency was registered with composite scintillator with a Ga content of x = 2.17 in the SCF part; however, the LY of this sample is more than 3 times lower than that of GAGG:Ce substrate (Figure 17a and Figure 18a).

Under γ-excitation of Gd_3_Al_5−x_Ga_x_O_12_:Ce SCFs/GAGG:Ce composite scintillators, the main peaks in the PHS correspond to the total absorption of 662 keV γ-rays (Figure 17b). The additional peak at 32 keV relates to the low-energy line of ^137^Cs source.

Quite-similar positions of the main peaks are observed in Figure 17b and Figure 18b for all composite scintillators and GAGG2.5:Ce SC, because γ-rays excite mainly the substrates in Gd_3_Al_5−x_Ga_x_O_12_:Ce SCFs/GAGG2.5:Ce epitaxial structures, and contribution of SCF scintillators to the total LY of composite scintillators is insignificant.

**Figure 19:**
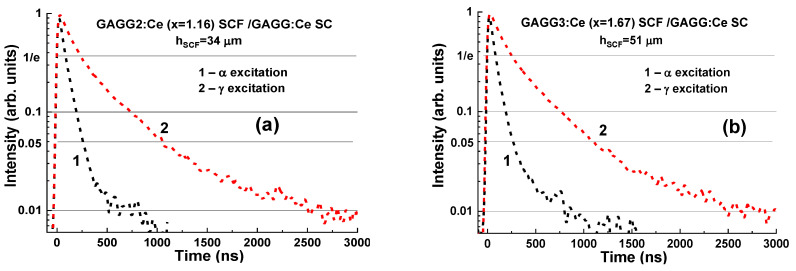

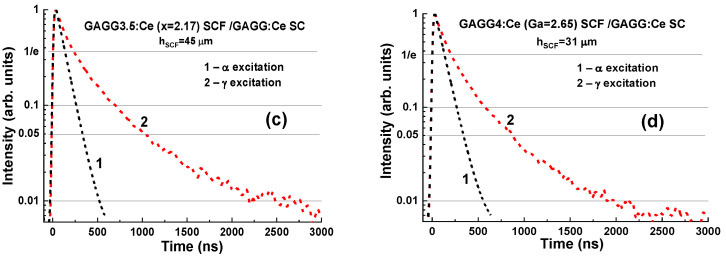
Scintillation decay curves of Gd_3_Al_5−x_Ga_x_O_12_:Ce SCF/GAGG2.5:Ce composite scintillators containing SCF with Ga content x = 1.16 (**a**); 1.67 (**b**); 2.17 (**c**); and 2.65 (**d**) grown onto GAGG:Ce 2.5 substrates under α-particle (curves 1) and γ-ray (curves 2) excitations [29].

**Figure 20:**
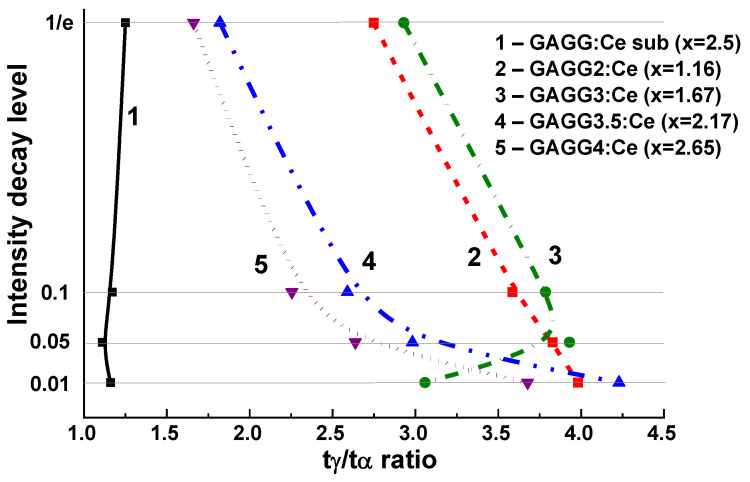
Plot of t_γ_/t_α_ ratio on scintillation decay to 1/e, 0.1, 0.05, 0.01 levels for GAGG2.5:Ce substrate (1) and Gd_3_Al_5−x_Ga_x_O_12_:Ce SCF/GAGG2.5:Ce composite scintillators with different Ga contents x (2–5) [22].

The difference in the scintillation decay curves of the bulk and film components of composite scintillators is determined by the analysis of decay curves in a broad decay intensity range under α-particle and γ-ray excitation of GAGG:Ce substrates with different Ga content. We performed such an analysis for the GAGG2.5:Ce substrate and the GAGG3:Ce reference crystal with the same thickness of 0.9 mm for scintillation decay to levels 1/e, 0.1, 0.05, and 0.01 with excitation with α-particles ^239^Pu (5.5 MeV) and γ-rays ^167^Cs (662 KeV) radiation sources (Table 2). The deconvolution of the corresponding decay curves was performed using the approximation I = A_i_exp (−t/τ_i_) + const, and its results are presented in Table 2. To quantify the differences in decay curves at the 1/e, 0.1, 0.05, 0.01 levels, the t_α_/t_γ_ ratios for respective decay times were used (Table 3 and Figure 19 and Figure 20). As shown in Figure 19a,b, decay curves for both GAGG2.5:Ce and GAGG3:Ce crystals under the γ-quanta excitation are systematically faster than in the case of excitation with α-particles. This is the fundamental behavior of scintillation materials related to the specific interaction of α-particles and γ-quanta with the scintillator material with different cation content. This effect is quantified by the t_α/_t_γ_ ratios in GAGG2.5:Ce substrate and reference GAGG3:Ce crystal (Figure 20). As can be seen in this figure, the ratio t_α/_t_γ_ varies in the ranges 1.17–1.26 and 1.46–1.49, respectively, *and the value of this ratio is much higher in garnet crystals with high Ga content*. Meanwhile, taking into account the slower decay and smaller differences in the t_α/_t_γ_ ratio in GAGG2.5:Ce crystals, such compositions are more suitable in the development of composite scintillators based on the decay-curve differences of GAGG:Ce substrates and Gd_3_Al_5−x_Ga_x_O_12_:Ce (x = 1.16–2.615) SCFs (Figure 19).

The separation rate of the scintillation signal at the registration of α-particles and γ-rays may be improved in the epitaxial structures based on the SCFs and SCs of Gd_3_Al_5−x_Ga_x_O_12_:Ce garnets with different Ga content. Indeed, substantial differences in the scintillation decay are observed in Figure 19 in the four types of Gd_3_Al_5−x_Ga_x_O_12_:Ce SCF/GAGG2.5:Ce SC composite scintillators at x = 1.16 (a), 1.67 (b),2.17 (c), and 2.65 (d) under α-particles and γ-quanta excitations. Figure 20 demonstrates the t_α_/t_γ_ ratios for scintillation decay to the 1/e, 0.1, 0.05, and 0.01 levels for these composite scintillator types (curves 1–4), respectively.

Decay time values to the mentioned levels for four samples of composite scintillators under α-particles and γ-quanta excitations are presented in Table 3. Note here that the decay profiles of Gd_3_Al_5−x_Ga_x_O_12_:Ce SCF scintillators under α-particle excitation *show weak correlation with Ga content in the 1.16–2.165 range*. Therefore, the observed significant differences in scintillation decay of Gd_3_Al_5−x_Ga_x_O_12_:Ce SCFs under α-particle excitation (Figure 19) in comparison with bulk crystals (Figure 11) are *mainly due the Pb^2+^ doping and recharging part of Ce^3+^ ions to Ce^4+^ state in SCF scintillators* [22,62,63,64]. This causes the scintillation decay acceleration in doubly doped Ce^4+^-Pb^2+^ SCF scintillators. Furthermore, such a phenomenon *enables the separation of the decay kinetics of SCF scintillators even in the case of* *similar Ga content in the x = 2.17–2.615 range (*Figure 19*, c and d) with Ga concentration x = 2.5 in the substrate*. These results are well consistent with the previously published data on the scintillation properties of SCFs and SCs of Gd_3_Al_5−x_Ga_x_O_12_:Ce garnets [22].

An efficient separation of the signals from SCF and SC substrate at 1/e, 0.1, and 0.05 intensities (Figure 19 and Figure 20) can be obtained *for all the developed types of composite scintillators in the whole 100–3000 ns time range*. Furthermore, the largest differences in the scintillation decay curves of composite scintillators under α- and γ-excitations were observed between the 0.1 and 0.01 levels. The best α/γ separation was observed with Gd_3_Al_5−x_Ga_x_O_12_:Ce/GAGG2.5:Ce epitaxial structures at x = 1.16 and 1.67 (Figure 20, curves 2 and 3). The t_γ_/t_α_ ratio was in the 2.75–4 range at scintillation decay to 1/e, 0.1, and 0.05 levels. Meanwhile, *taking into account the high LY of SCF scintillator and a high t_γ_*/*t_α_ ratio for SCF and SC parts of* *composite scintillators equal to 1.8–4.2 at 1*/*e, 0.1, and 0.05 levels of the intensity decay (*Figure 20*, curve 4),* *the best scintillation figure-of-merit was observed for* *Gd*_3_*Al*_2.83_*Ga*_2.17_*O*_12_*:Ce:Ce SCF*/*GAGG2.5:Ce epitaxial structure*.

#### 3.2.3. Scintillating Screens Based on the LPE-Grown Ce^3+^-Doped Tb_3_Al_5_O_12_ and Tb_3−x_Gd_x_Al_5−y_Ga_y_O_12_ Garnets

Cation-composition engineering opens new possibilities in the development of scintillators based on SCFs of mixed garnets. Crystallization of SCFs on different substrates using different fluxes enables optimization of excitation transfer deficiency to activators to ensure a high scintillation efficiency of composite scintillators. The bandgap, and position of 5d energy levels of Ce^3+^ ion in the bandgap, may be controlled by a combination of Gd^3+^, Tb^3+^, and Ga^3+^ cations (see Figure 2). These corresponding changes in crystal field strength are caused by Gd^3+^ and Tb^3+^ cation substitution in dodecahedral positions and Ga^3+^ ions by Al^3+^ cations both in the tetrahedral and octahedral positions in the garnet lattice.

**Figure 21:**
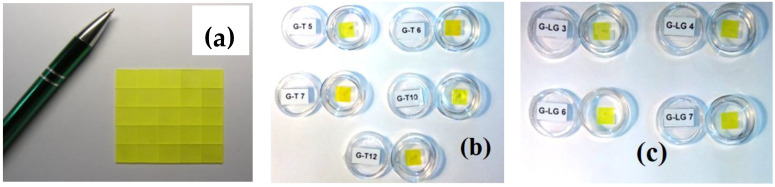
GAGG:Ce substrates (**a**), TbAG:Ce SCF/GAGG:Ce SC (**b**), and LuAG:Ce SCF/GAGG:Ce SC (**c**) composite scintillators [20,21].

**Figure 22:**
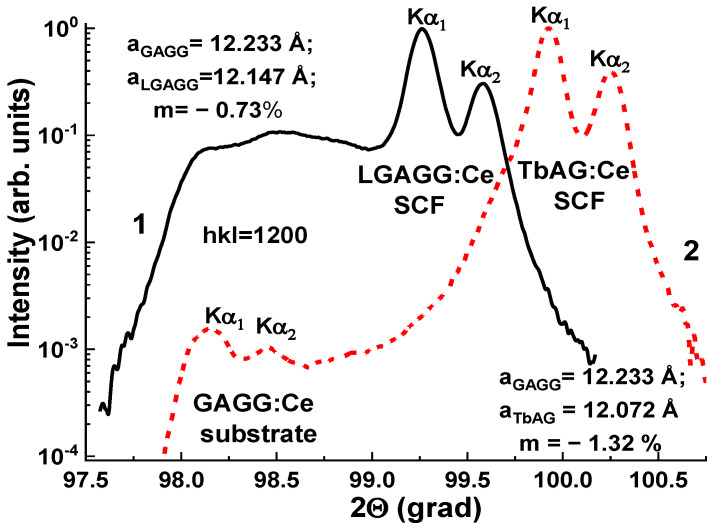
XRD patterns of LGGAG:Ce SCF/GAGG:Ce SC (1) and TbAG:Ce SCF/GAGG:Ce SC (2) epitaxial structures [20,21].

Following the successful tests of GAGG:Ce SCF/GAGG:Ce SC composite scintillators, SCFs of LuAGG:Ce and TbAG:Ce garnets were crystallized (Figure 21) [20,21] by the LPE method using the PbO-B_2_O_3_ flux both on “traditional” YAG and Gd_3_Ga_2.5_ Al_2.5_O_12_ (GAGG) substrates with the lattice constants of 12.01 and 12.232 Å, respectively, and their structural properties were determined as well. The measured mismatch of lattice constants for these composite scintillators was −0.73% and −1.32%, respectively (Figure 22), and small enough to obtain SCFs of a good optical quality. Meanwhile, the crystallization of LuAGG:Ce and TbAG:Ce SCFs on GAGG:Ce substrates with x = 3.0 failed due to a large mismatch over 2%. According to XRD measurements, the lattice constant mismatch (m) between TbAG:Ce SCF and YAG and GAGG substrates was +0.53–0.56% and −1.29%, respectively (Table 4). TbAG SCF crystallization on YAG and GAGG substrates with such a large mismatch is complicated due to the formation of a transitional zone in the form of a solid solution at the film–substrate interface reducing this mismatch.

TbAG:Ce SCFs crystallized on YAG and GAGG substrates possess 30% higher LY (Table 4) and exceptionally low level of phosphorescence compared to YAG:Ce and LuAG:Ce SCFs [68]. These SCFs are also characterized by relatively fast scintillation decay in the range of 0–1000 ns. Therefore, TbAG:Ce SCFs based on GAGG:Ce substrates are very promising for microtomographic detector screens, as well as a suitable component for composite scintillator engineering.

**Figure 23:**
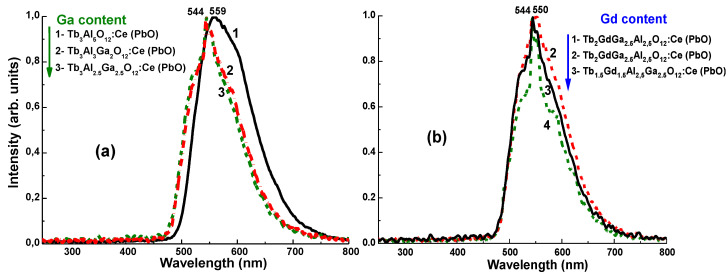
Normalized CL spectra of Tb_3_Al_5−y_Ga_y_O_12_Ce (PbO) (**a**), Tb_5−x_Gd_x_Al_2.5_Ga_2.5_O_12_:Ce (PbO) (**b**) SCFs with different x and y (see figure legend) [68,69].

Corresponding changes in crystal field strength caused by substitution of Gd^3+^ by Tb^3+^ in dodecahedral positions and Ga^3+^ by Al^3+^ cations both in the tetrahedral and octahedral positions in the garnet lattice result in the blue- or redshift of the Ce^3+^ luminescence spectrum in Tb_3−x_Gd_x_Al_5−y_Ga_y_O_12_:Ce garnets as Ga^3+^ (Figure 23a) or Gd^3+^ (Figure 23b) contents increase. A complex cascade of Gd^3+^→Tb^3+^→Ce^3+^→Tb^3+^ energy transfer is observed in Tb_3−x_Gd_x_Al_5−y_Ga_y_O_12_:Ce garnets, with a high concentration of Gd^3+^ and Tb^3+^ cations. The reason for this transfer is the overlap of Gd^3+^ and Tb^3+^ emission bands and Ce^3+^ ion absorption bands in the UV range, as well as Ce^3+^ ion emission bands and Tb^3+^ absorption bands in the blue range.

For this reason, optimization of Gd^3+^, Tb^3+^, and Ga^3+^ contents in Tb_3−x_Gd_x_Al_5−y_Ga_y_O_12_:Ce garnet at x = 1.5 and y = 2–2.5 provides a significant improvement in the film scintillation efficiency due to more favorable excitation energy-transfer conditions caused by changes in the band gap and Ce^3+^ energy structure.

**Figure 24:**
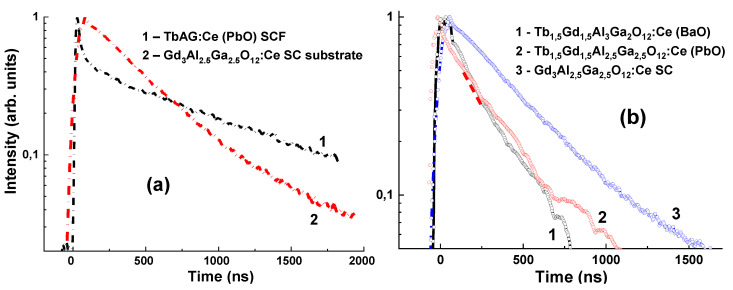
The normalized scintillation decay of Tb_3_Al_5_O_12_:Ce (PbO) SCF (**a**) Tb_1.5_Gd_1.5_Al_2_Ga_3_O_12_:Ce (BaO) and Tb_1.5_Gd_1.5_Al_2.5_ Ga_2.5_O_12_:Ce (PbO) in comparison with that in bulk Gd_3_Al_2.5_Ga_2.5_O_12_:Ce SC (**b**, curve 3) [68,69].

Tb_3−x_Gd_x_Al_5−y_Ga_y_O_12_:Ce (PbO) SCFs demonstrate a very high structural quality, while the SCFs grown from the BaO flux possess excellent scintillation properties, but the structure quality is slightly lower due to the high flux density. It is very important that the scintillation decay kinetics of the Tb_1.5_Gd_1.5_Al_2.5_Ga_2.5_O_12_:Ce (PbO) and, especially, Tb_1.5_Gd_1.5_Al_3_Ga_2_O_12_:Ce (BaO) SCFs, is notably faster by at least 2 times in the range of 0–2 µs as compared to GAGG:Ce crystal (Figure 24). These SCFs and high-quality Gd_3_Al_2.5_Ga_2.5_O_12_:Ce crystals can be used in composite scintillators for simultaneous registration of different components of ionization fluxes. 

**Figure 25:**
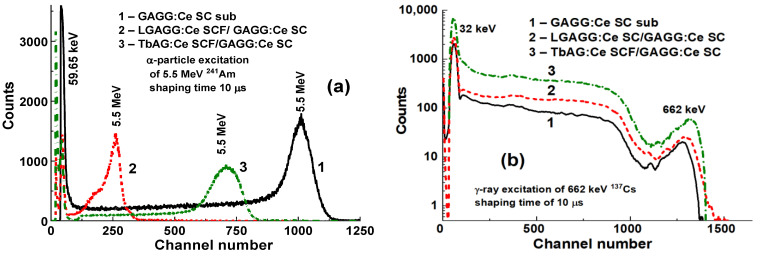
PHS of LGAGG:Ce SCF/GAGG:Ce SC (2), TbAG:Ce SCF/GAGG:Ce SC (3), and GAGG:Ce substrate (1) measured in a time range of 10 µs at excitation with α-particle (**a**) and γ (**b**).

The results obtained on composite scintillators based on LuAG:Pr and LuAG:Sc substrates indicate that the simultaneous excitation of the substrate and film by γ-quanta significantly affects their pulse height spectra (Figure 7b and Figure 9b) and scintillation decay kinetics (Figure 8 and Figure 10). In contrast, in LGAGG:Ce SCF/GAGG:Ce SC and TbAG:Ce SCF/GAGG:Ce SC composite scintillators, α-particles excite only SCFs, which is indicated by the different locations of the main peaks in the PHS spectra (Figure 25a). Under excitation by γ-quanta, the main peaks of both composite scintillators and GAGG:Ce substrate almost coincide, which means that the substrate in these composites is mainly excited (Figure 25b).

**Figure 26:**
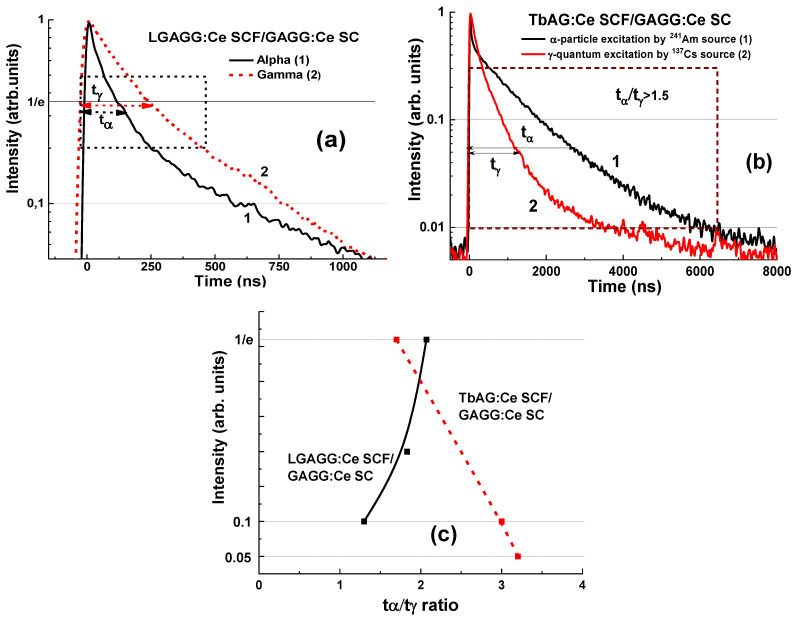
Scintillation decay of LGAGG:Ce SCF/GAGG:Ce SC (**a**) and TbAG:Ce SCF/GAGG:Ce SC (**b**) composite scintillators under excitation with α-particle (1) and γ-quantum (2). (**c**)—t_γ_/t_α_ ratio for different levels of scintillation decay for these two types of composites [20,21].

A large difference in scintillation decay is observed under α-particles and γ-quanta excitations (Figure 26a,b) quantified by the t_α_/t_γ_ ratio, which is 1.3–2.07 and 1.7–3.2 as luminescence decays from 1/e to 0.1 and 0.05 levels, respectively (Figure 26c). For the LGAGG:Ce SCF/GAGAG:Ce SC composite scintillator, the scintillation signals from the SCF and substrate can also be separated with a high t_α_/t_γ_ ratio in a narrow time range of 0–500 ns, but in a slightly narrower range of luminescence intensity between 1/e and 0.1 (Figure 26a). TbAG:Ce SCF/GAGG:Ce SC composite scintillator has superior properties compared to the former epitaxial structure due to the higher t_α_/t_γ_ in a wide time range of 0–6000 ns as luminescence intensity decreases from 1/e to 0.01 (Figure 26c). These results certify that both these types of composite scintillators are capable of discriminating successively between α-particles and γ-quanta in mixed ionization beams.

### 3.3. Composite TL Detectors

Composite scintillators disclosed in the previous sections showed the ability to simultaneous registration of components of mixed ionizing fluxes by their scintillation decay at the excitation with α-particles and γ-quanta. Meanwhile, β-particles with energies in a wide range from keV to MeV in mixed ionizing beams were hardly detected in this way. The use of composite scintillators operating in in situ mode is also complicated under registration of low doses of radiation during long-term exposure.

Another option is a registration of thermoluminescence curves (TL) from an SCF and a substrate of a composite material produced by the LPE method. Such TL detectors were based on Ce^3+^-doped LuAG and YAG garnets and their capability to simultaneously detect α- and β-particles, and X-ray or γ-quanta was verified [23,24].

#### 3.3.1. LuAG:Ce SCF/YAG:Ce and YAG:Ce SCF/LuAG:Ce SC Composite TL Materials

**Figure 27:**
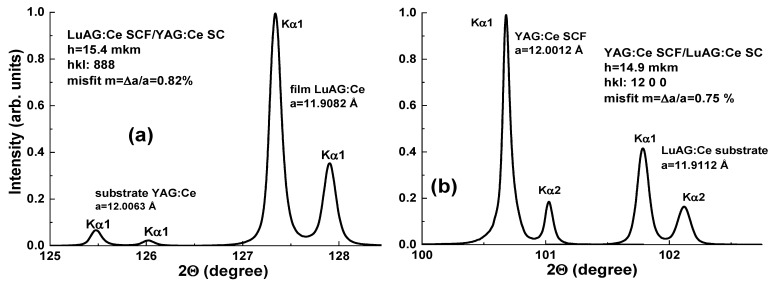
XRD pattern of LuAG:Ce SCF/YAG:Ce SC (**a**) and YAG:Ce SCF/LuAG:Ce SC (**b**) epitaxial structures [24].

The first prototypes of composite TL materials were created based on YAG:Ce SCF/LuAG:Ce SC and LuAG:Ce SCF/YAG:Ce SC epitaxial structures. The fabrication of such structures by the LPE method was technologically challenging because a misfit in the SCF and substrate lattice constants, which was about of 0.8% for the both composites (Figure 27), is less than the border condition value of ±1% for SCF growth of garnet compounds [23].

The chosen contents of these composites were based on previous observations of TL properties of SCFs and substrates of these garnets, namely, a large difference in growth temperature, the gas atmosphere composition, and types of defects and admixtures. Ce^3+^ ions are typically served as hope trapping centers, while TL peaks in Ce^3+^-doped films and crystals of garnet compounds under study correspond to electron trapping centers. Such centers in garnets can be oxygen vacancies and their aggregates with other defects, in particular, substitutional defects related to incorporation of Pb ions in SCFs of these materials.

The primary task was to compare the main TL peaks from YAG:Ce and LuAG:Ce SCFs and crystals under excitation by α- and β-particles. α-particles of an ^241^Am source (5.5 MeV) should be completely absorbed in the SCF part of the composite materials, while β-particles of the ^90^Sr + ^90^Y source with an average energy of 1.1 MeV can be absorbed by 1.3 mm-thick YAG:Ce and 0.8 mm-thick LuAG:Ce substrates. Hence, TL signals from the SCFs and substrates correspond mainly to the registered α-particles and β-particles, respectively.

**Figure 28:**
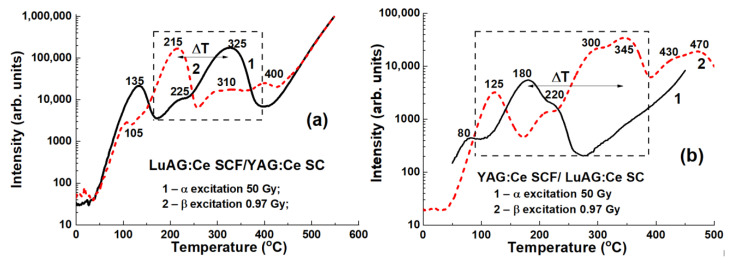
TL glow curves of LuAG:Ce SCF/YAG:Ce SC (**a**) and YAG:Ce SCF/LuAG:Ce SC (**b**) TL composite materials after irradiation with α- (1) and β- (2) particles [23].

The main peaks for LuAG:Ce SCF/YAG:Ce SC structures are registered at temperatures of 325 °C and 215 °C after irradiation with α- and β-particles, respectively (Figure 28a), while for YAG:Ce SCF/LuAG:Ce structure the corresponding peaks are at 180 °C and 345 °C, respectively (Figure 28b). Therefore, the difference ΔT between the main TL peaks used as a discrimination measure of these two types of particles is 110 °C and 165 °C for these types of composites, respectively.

#### 3.3.2. Lu_3−x_Gd_x_AG:Ce SCF/YAG:Ce SC Composite TL Materials

**Figure 29:**
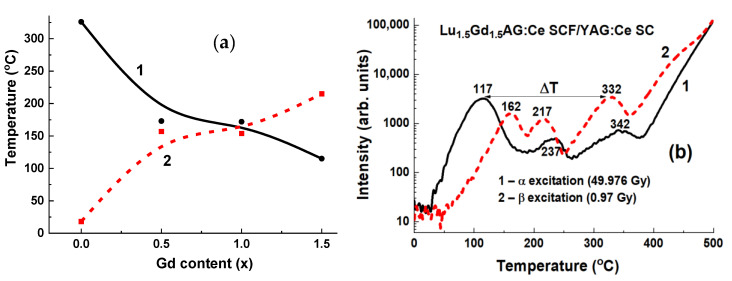
(**a**)—Dependence of the main Lu_3−x_Gd_x_Al_5_O_12_:Ce SCF/YAG:Ce SC TL peak location on Gd content after excitation with α-particles (1), and the temperature interval between the main TL peaks of the Lu_3−x_Gd_x_Al_5_O_12_:Ce SCF film and YAG:Ce substrate after excitation with α- (^241^Am) and β- (^90^Sr + ^90^Y) particles (2). (**b**)—glow curves of these composite materials after excitation with α- (1) and β-particles (2) [23].

Following the successful development of LuAG:Ce/YAG:Ce TL composite materials, LuAG:Ce films in them were modified by adding Gd. In the Lu_3−x_Gd_x_Al_5_O_12_:Ce SCF/YAG:Ce SC structures with x = 0–1.5 (Figure 29), the difference between the main TL peak positions after irradiation with α- and β-particles gradually increases with the increase in Gd content (see [24] for TL details). The obtained difference (ΔT) in the main TL peak locations increased compared to the LuAG:Ce SCF/YAG:Ce SC structures from 110 to 215 °C.

Therefore, composite materials capable of registering simultaneously α- and β-radiation in mixed ionizing beams using the phenomenon of TL were developed. The developed epitaxial structures are the prototypes for constructing a new generation of composite TL detectors based on various oxide compounds crystallized using the LPE method.

## 4. Concluding Remarks

The paper reviews three different approaches to development of composite scintillators and TL materials based on the garnet epitaxial structures for the simultaneous detection of particles and quanta in mixed ionizing beams. The **first approach** deals with choosing the dopants in SCFs and a substrate of the same crystalline host, namely, LuAG garnet, which provide different scintillation properties. The **second approach** is based on the use of various types of Ce^3+^-doped SCFs and crystals of mixed garnets, possessing different scintillation properties. The **third approach** is based on the difference in TL properties of SCFs and substrate of simple or mixed garnets doped with Ce^3+^ ions.

The possibility of crystallizing (Gd,Tb,Lu)_3_(Al,Ga)_5_O_12_:Ce SCFs of mixed garnets by the LPE growth method onto YAG and GAGG substrates was demonstrated using PbO and BaO fluxes. Such developed SCF scintillators can be used in microtomographic devices as scintillation screens with X-ray quanta absorption ability. At the same time, such SCF scintillators can also be used in monitoring of α- or/and β-particles in mixed radiation fluxes.

Table 5 summarizes the developed composite scintillators and their scintillation properties.

Composite scintillators based on SCFs and crystals of Ce^3+^-doped mixed garnets possess higher t_α_/t_γ_ and higher LY in comparison with counterparts based on doped SCFs and crystals of LuAG garnet. LY in the developed TbAG:Ce and (Gd,Tb)(Al,Ga)O_12_:Ce SCF scintillators is the highest among the known garnet SCFs obtained from PbO-based fluxes and show a very low afterglow level in the scintillation response. The highest t_α_/t_γ_ ratio of up to 3 in the time interval of 450–3700 ns at the scintillation intensity decay level of 0.05 is registered in Tb_3_Al_5_O_12_:Ce SCF/Gd_3_Al_2.5_Ga_2.5_O_12_:Ce composite scintillator. The highest LY and the best separation of the decay curves under excitation by α-particles and γ-quanta among all the studied composition was achieved for Gd_3_Al_5−x_Ga_x_O_12_:Ce (x = 1.15–2.67) SCF/GAGG2.5:Ce SC epitaxial structures, where the t_γ_/t_α_ reached 4.2 in the 0–3000 ns time range at scintillation decay from 1/e to 0.01 levels. *This is the best scintillation figure-of-merit among all the developed types of composite scintillators based on SCFs and crystals of mixed garnets.* Therefore, such a type of developed composite scintillator can be successfully applied for simultaneous registration of α- and β-particles and γ-quanta in mixed radiation beams.

Prototypes of TL composite materials based on epitaxial structures of garnet compounds comprising SCFs and crystals of YAG:Ce and LuAG:Ce garnets were developed also using the LPE method. It has been shown that such composites can be used for detection of α- and β-particles using the differences between TL glow curves recorded from the SCFs and substrates. Among all the developed composite TL materials based on SCFs and crystals of garnet compounds, the most efficient registration of α- and β-particles is achieved with the YAG:Ce SCF/LuAG:Ce SC and Lu_1.5_Gd_1.5_AG:Ce SCF/YAG:Ce SC epitaxial structures where the temperature difference between the main TL peaks corresponding to the detection of α- and β-particles is equal to 165 and 215 °C, respectively.

Meanwhile, there is still room for the development of new types of composite scintillators and TL detectors with enhanced scintillation and TL properties. Such new types of composite detectors can be created on the basis of materials with more suitable scintillation and TL characteristics in comparison with the above-mentioned garnet compounds. Namely, Lu_2_SiO_5_ (LSO) orthosilicate, (Lu,Gd)_2_Si_2_O_5_ (LGPS) pyrosilicate, LuAlO_3_ (LuAP) perovskite, as well as CdWO_4_ (CWO) tungstate hosts have significantly high density and effective atomic number in comparison with Y-, Lu-, and Gd-based garnets [12,43,44]. For this reason, the Ce^3+^-doped LSO, LGPS, and LuAP crystals as well as undoped CWO crystal are very promising materials for producing the respective substrates at developed composite scintillators and TL detectors. The Ce^3+^- and Bi^3+^-doped SCFs of Y_2_SiO_5_ (YSO) orthosilicate, (Y,Gd)_2_Si_2_O_12_ (YGdPS) pyrosilicate, YAlO_3_ (YAP), and GdAlO_3_ (GAP) perovskites and their solid solutions, as well as Bi^3+^-doped CWO SCFs, are also suitable compounds for the creation of film components of composite scintillators and TL detectors on their base [74,75,76,77,78,79,80,81,82,83,84].

Furthermore, the possibility of fabricating by the LPE method the advanced multilayered composite scintillators and TL detectors based on the films and crystals of all the above-mentioned oxide compounds needs technological and experimental confirmation. In future, we plan to fabricate new types of multilayered scintillation and TL materials based on several (two and more) SCFs and single crystals of doped garnets, ortho-, and pyrosilicates, perovskites, and tungstates, with enhanced scintillation properties as compared to all well-known analogues, grown by the LPE method, for the simultaneous registration of the different components of the mixed radiation beams, including α- and β-particles, X-rays, and γ-quanta with various energies and penetration depth in the mentioned oxide materials.

## Figures and Tables

**Table 1:** Selected properties of materials for garnet composite scintillators. The data were collected from the producer websites [40,41,42,43,44] (literature data in brackets may differ from the data provided by producers).

Parameter	YAG:Ce	LuAG:Ce	LuAG:Pr	LuAG:Sc	GAGG:Ce
Density (g/cm^3)^	4.57	6.73	6.73	6.73	6.63
Effective atomic number	74	58.9	62.9	61	54.4
Wavelength of max. emission (nm)	550	535	310	280	520
Decay constant (ns)	70	70	20 (19–28)	245–610	50–150
Photon yield (ph/MeV)	30 × 10^3^	25 × 10^3^	15–18 × 10^3^	22.5 × 10^3^	40–60 × 10^3^
LY_α_/LY_γ_ in the range 0.5–10 µs		0.1–145	0.31–0.34	0.38–0.42	0.19–0.2
Energy resolution (%)	6.7	5.5–7	<5	7	6.68

**Table 2:** Real compositions of Gd_3_Al_5−x_Ga_x_O_12_:Ce SCF, type of substrate, h—SCF thickness, *T_g_*_—_SCF growth temperature, f_g_—SCF growth rate, LY—light yield under α-particle excitation from ^239^Pu and ^241^Am sources with respect to the standard YAG:Ce SCF sample with an LY of 2.65 photons/KeV [20,22].

No SCF and SC	Nominal SCF Content in Melt-Solution	Substrate Type	Real SCF Compositions	h, µm	*T_g_*, °C	f_g_, μm/min	LY, % Pu^239^ (12 μs)	LY, % Am^241^ (3 μs)
YAG:Ce	Y_3_Al_5_O_12_:Ce	YAG	Y_3_Al_5_O_12_:Ce	54			100	
GAGG2.5:Ce	Gd_3_Al_2.5_Ga_2.5_O_12_:Ce	–	–	900			340	100
GAGG3:Ce	Gd_3_Al_2_Ga_3_O_12_:Ce	–	–	900			320	
GAGG2:Ce	Gd_3_Al_3_Ga_2_O_12_:Ce	GAGG2.5:Ce	Gd_3.038_Ce_0.005_Pb_0.001_ Al_3.792_Ga_1.162_O_12_	34	970	1.13	59	18.4
GAGG3:Ce	Gd_3_Al_2_Ga_3_O_12_:Ce	GAGG2.5:Ce	Gd_3.08_Ce_0.003_Pb_0.077_ Al_3.141_Ga_1.67_O_12_	51	1000	1.15	42	27.1
GAGG3.5:Ce	Gd_3_Al_1.5_Ga_3.5_O_12_:Ce	GAGG2.5:Ce	Gd_3.28_Ce_0.009_Pb_0.056_ Al_2.528_Ga_2.17_O_12_	45	974	0.56	32	29.9
GAGG4:Ce	Gd_3_AlGa_4_O_12_:Ce	GAGG2.5:Ce	Gd_3.29_Ce_0.017_Pb_0.012_ Al_2.615_Ga_2.615_O_12_	36	985	1.2	31	17.9

**Table 3:** t_α_ and t_γ_ values of scintillation decay to 1/e, 0.1, 0.05, 0.01 intensities of Gd_3_Al_5−x_ Ga_x_O_12_:Ce SCF/GAGG2.5:Ce SC composite scintillators with varied Ga concentrations in SCF under α-particle and γ-ray excitation.

	GAGG2.5:Ce Substrate		GAGG2:Ce SCF/GAGG:Ce Sub		GAGG3:Ce SCF/GAGG:Ce Sub		GAGG3.5:Ce SCF/GAGG:Ce		GAGG4:Ce SCF/GAGG:Ce Sub	
	t_α_, ns	t_γ_, ns	t_α_, ns	t_γ_, ns	t_α_, ns	t_γ_, ns	t_α_, ns	t_γ_, ns	t_α_, ns	t_γ_, ns
1/e	390	310	87	2239	86	252	129	235	133	208
0.1	925	790	207	742	205	776	270	700	275	583
0.05	1300	1170	280	1072	287	1128	345	1029	355	888
0.01	2875	2465	638	2540	835	2554	533	2254	590	1976

**Table 4:** Misfit between SC and SCF lattice parameters, *m*, CL band maximum, *λ_max_*, the ratio of decay times at different levels of scintillation decay, and relative ligÅht yield of SCFs.

SCF	SC	*m*,%	*λ_max_*, nm	t_1/e_/t_1/20_, ns	LY,%
LuAG:Ce	YAG	−0.82	509	53	205
LuAG:Pr	YAG	−0.8	305	17	79
LuAG:Sc	YAG	−0.8	280	245; 390	96
Lu_1.5_Gd_1.5_Al_5_O_12_:Ce	YAG	+0.02	548	50	86
Lu_1.5_Gd_1.5_Al_2.75_Ga_2.25_O_12_:Ce	GAGG	−0.73	519	51/130	145
TbAG:Ce	YAG GAGG	+0.55 −1.29	555 560	242/1645 306/1795	253–264 195
Tb_1.5_Gd_1.5_Al_2.5_Ga_2.5_O_12_:Ce (PbO)	GAGG	−0.12	543	333/990	380
Tb_1.5_Gd_1.5_Al_3_Ga_2_O_12_:Ce (BaO)	GAGG	−1.30	543	228/728	380

**Table 5:** Comparison of scintillation properties of different composites materials based on garnet epitaxial structures.

Composite Scintillator	The Best Ratio t_α_/t_γ_	Intensity Level for the Best t_α_/t_γ_ Ratio	Optimal Value of Intensity	Time Interval (ns)
LuAG:Pr SCF/LuAG:Ce SC	3.6	0.1	0.03–0.4	0–700
LuAG:Sc SCF/LuAG:Ce SC	2.2	0.05	0.05–0.2	200–900
LuAG:Ce SCF/LuAG:Pr SC	1.1	0.05	0.05–0.2	60–320
Lu_2.85_Tb_0.15_AG:Ce SCF/LuAG:Pr SC	1.2	0.05	0.1–0.02	200–1500
Lu_1.75_Tb_0.3_AG:Ce SCF/LuAG:Pr SC	1	-	-	-
Lu_1.35_Tb_1.65_AG:Ce SCF/LuAG:Pr SC	1.4	0.1	0.05–0.2	250–1000
Lu_0.715_Tb_2.285_AG:Ce SCF/LuAG:Pr SC	4.2	0.07	0.05–0.36	100–3000
LuAG:Ce SCF/LuAG:Sc SC	2.7	0.05	0.02–0.2	250–110
LuAG:Pr SCF/LuAG:Sc SC	15.6	0.05	0.01–0.5	0–2500
TbAG:Ce SCF/GAGG:Ce SC	3	0.05	0.05–0.2	450–3700
LGAGG:Ce SCF/GAGG:Ce SC	2	1/e	0.2–0.5	50–500
GAGG/GAGG	4.2	0.01	0.01–0.1	600–2700

## Data Availability

Not applicable.
